# Use of hCG for luteal support in natural frozen–thawed blastocyst transfer cycles: a cohort study

**DOI:** 10.3389/fendo.2024.1391902

**Published:** 2024-08-14

**Authors:** Wen Wen, Na Li, Juanzi Shi, Hanying Zhou, Lijuan Fan

**Affiliations:** Assisted Reproduction Center, Northwest Women’s and Children’s Hospital, Xi’an, China

**Keywords:** luteal phase support, human chorionic gonadotropin, natural cycle, frozen-thawed transfer, live birth rate

## Abstract

**Introduction:**

In the realm of natural frozen-thawed embryo transfer (FET) cycles, the application of luteal phase support (LPS) is a prevalent practice, primarily due to its beneficial impact on reproductive outcomes. Among the various LPS medications, human chorionic gonadotropin (hCG) is one that exerts its function on both the corpus luteum and the endometrium.

**Objective:**

To evaluate the effect of hCG administration as LPS on reproductive outcomes in natural FET cycles.

**Methods:**

This study was a retrospective cohort analysis conducted at a tertiary care hospital. It included women who underwent natural FET treatment from January 2018 to December 2022. Participants were divided into the hCG LPS group and the non-hCG LPS group on the basis of whether they used hCG as LPS after blastocyst transfer. The primary outcome was the clinical pregnancy and live birth rates. The secondary outcomes included the early miscarriage rate (before 12^th^ gestational week) and total miscarriage rate.

**Results:**

A total of 4762 women were included in the analysis, and 1910 received hCG LPS and 2852 received no hCG LPS (control group). In the general cohort, the clinical pregnancy and live birth rates in the hCG LPS group were significantly lower than those in the control group (63.82% vs 66.41%, aOR 0.872, 95% CI 0.765–0.996, *P*=0.046; 53.98% vs 57.15%, aOR 0.873, 95% CI 0.766–0.991, *P*=0.035, respectively). The early miscarriage and total miscarriage rates were similar between the two groups. In a subgroup analysis, in women who received an hCG trigger, there was no significant difference in the clinical pregnancy rate or live birth rate between the two groups. However, in women who ovulated spontaneously, the clinical pregnancy and live birth rates in the hCG LPS group were significantly lower than those in the control group (60.99% vs 67.21%, aOR 0.786, 95% CI 0.652–0.946, *P*=0.011; 50.56% vs 57.63%, aOR 0.743, 95% CI 0.619–0.878, *P*=0.001, respectively).

**Conclusion:**

Among women undergoing natural cycle frozen–thawed blastocyst transfer, hCG LPS is associated with lower clinical pregnancy and live birth rates. Additionally, the adverse effect of hCG LPS is more pronounced in women who ovulate spontaneously.

## Introduction

In frozen–thawed embryo transfer (FET) treatment, natural cycles refer to the cycles in which follicles develop spontaneously, and subsequently ovulate and shift to the corpus luteum. The corpus luteum produces progesterone and estrogen, which are essential for embryo implantation and subsequent pregnancy. Because of the presence of the corpus luteum, there is a less demand for exogenous administration of luteal support in natural cycles. Although there is little evidence to support the benefit of luteal phase support (LPS) in natural cycles, LPS is commonly used in practice.

Progesterone and human chorionic gonadotropin (hCG) represent the two most frequently utilized luteal phase support (LPS) medications. These can be administered either independently or in conjunction during natural cycles. Notably, hCG serves as one of the earliest signals released by the embryo and detected by the mother, an occurrence documented from the fertilization stage (2PN) within embryonic culture mediums (1, 2). The hCG operates on both the corpus luteum and the endometrium. It effectively regulates multiple metabolic pathways within the decidua, thereby contributing to endometrial receptivity. The hCG has been found to significantly inhibit the intrauterine insulin-like growth factor binding protein-1 (IGF-BP-1) and the macrophage colony-stimulating factor (M-CSF). Conversely, it significantly stimulates the leukemia inhibitory factor (LIF), the vascular endothelial growth factor (VEGF), and the matrix metalloproteinase-9 (MMP-9) (3–5). hCG prompts the corpus luteum to produce progesterone continuously via mimicking LH pulsatility. The mechanism of this process is that hCG binds to the LH receptor of granulosa cells, and subsequently leads to more potent activation of the cyclic AMP protein kinase A pathway, which stimulates progesterone production (6). Studies *in vitro* have suggested that hCG promotes longevity of the corpus luteum via increasing levels of antiapoptotic BCL-2 and decreasing proapoptotic Bax (7). In addition to the effects of hCG on the corpus luteum, hCG may provide a signal regarding future embryo implantation to the endometrium, foster growth and differentiation of trophoblast cells, and establish placental villous structures (8). The biosynthesis of hCG begins early in embryonic development. Therefore, hCG is detected in relatively high concentrations in the uterine cavity prior to implantation, and exogenous administration of hCG during *in vitro* fertilization may mimic the local effects of hCG when fertilization occurs naturally (9). Consequently, many researchers originally believed that hCG should be the primary choice for LPS.

However, hCG LPS was reported as non-beneficial in a recently published meta-analysis, which included only two studies examining the use of hCG as the sole regimen for LPS in natural FET cycles (10). These two studies were both conducted by Lee et al. and only cleavage embryos were transferred. Their first study (11) was a retrospective cohort study in which women in the LPS group received two doses of 1500 IU hCG on the day of FET and 6 days later. They found that LPS with hCG did not increase the clinical pregnancy rate. The second study was a placebo-controlled, randomized, controlled trial (RCT), which included 450 women and they used the same regimen for LPS (12). These authors also found a comparable outcome in the LPS and the placebo groups.

In natural FET cycles, the effect of hCG may be related to the dosage and timing of hCG injection. T_max_ is 12 h and T_1/2_ is 23 h for hCG. Therefore, the difference in the dosage and timing of hCG injection may result in different effects on reproductive outcomes. We speculate that the use of continuous hCG injection after embryo transfer may be optimal. Advances in embryo culture media have led to exceeding blastocyst-stage embryo transfer over cleavage-stage embryo transfer. Blastocyst-stage embryo transfer is associated with better pregnancy outcomes than cleavage-stage embryo transfer (13). The effect of hCG LPS on natural cycle FET of blastocyst-stage embryos is unclear.

In this study, we aimed to evaluate the effects of continuous injection of hCG in natural FET. We conducted a retrospective study in women who had blastocysts transferred. We hypothesized that the use of hCG in natural FET as LPS is associated with increased clinical pregnancy and live birth rates.

## Materials and methods

### Study design and patients

This retrospective cohort study included all patients from a single center who underwent their first natural blastocyst FET cycle from January 2014 to December 2022. The exclusion criteria included: (i) cycles with letrozole or HMG for ovarian stimulation; (ii) female age at FET >35 years old; (iii) body mass index >30 kg/m^2^; (iv) endometrial thickness at FET <7 mm; (v) with an abnormal uterine cavity or untreated hydrosalpinx; (vi) recurrent implantation failure and recurrent pregnant loss; and (vii) PGT cycles. Data were extracted from electronic medical records. This study was approved by the Ethics Committee of Northwest Women’s and Children’s Hospital (No. 2022007).

### Monitoring of ovulation in the natural cycle

Women attended the clinic for ultrasound monitoring of follicular development from the 8^th^ day of menstruation. An hCG trigger (chorionic gonadotropin for injection; Lizhu, China) was provided for ovulation when the leading follicle reached 18 mm for longer than 2 days and transvaginal ultrasound showed anovulation. If the follicle fails to rupture within 2 days following trigger injection, the 36th hour post-injection will be deemed as the day of ovulation. Similarly, in cases where an unruptured follicle is observed post-trigger injection, the 36th hour following injection will also be considered as the day of ovulation. If ovulation was in doubt, further blood serum progesterone concentrations needed to be measured. Serum progesterone concentrations ≥3 ng/ml were considered manifestation of ovulation.

### Frozen–thawed blastocyst transfer

Blastocyst-stage embryos were thawed and transferred on the 5^th^ day after ovulation. A maximum of two blastocyst-stage embryos were allowed. Blastocyst evaluation was performed according to Gardner’s grading system.

### Luteal support

All women were administered oral progesterone (10 mg tid; Duphaston, Abbot, USA) and vaginal progesterone (90 mg qd, Crinone^®^ 8%; Merck Serono, Switzerland) for luteal support from the day of FET. In the hCG group, women received continuous hCG injection (2000 IU im qd; Lizhu, China) for 5 days from the day of blastocyst transfer. The addition of continuous hCG injection depended on the patients’ and physicians’ preferences. In both groups, oral progesterone and vaginal progesterone were continued until the day of pregnancy testing. If clinical pregnancy was diagnosed by ultrasound at 4 to 5 weeks after the blastocyst transfer, the LPS regimen was continued until 10 weeks of gestation.

### Definitions of pregnancy outcomes

Biochemical pregnancy was diagnosed only by the detection of serum beta hCG concentrations >50 mIU/ml at the 13^th^ day after FET. Miscarriage was defined as spontaneous loss of a clinical pregnancy before 22 completed weeks of gestation, and early miscarriage was defined as miscarriage before 12 weeks of gestational age. Preterm birth was defined as birth that occurred after 22 weeks and before 37 completed weeks of gestational age (14).

### Statistical analyses

Baseline characteristics are shown as the mean ± standard deviation and count (%), as appropriate. Categorical variables are presented as the proportion and percentage of the total. Comparison of continuous variables between the groups was performed using Student’s t-test or the Mann–Whitney U-test depending on the normality of the distribution. Fisher’s exact test was used to compare categorical variables. A multivariate regression analysis was used to assess the association between hCG LPS and various pregnancy outcomes while adjusting for female age, endometrial thickness, number of blastocysts transferred, hCG trigger administration and good quality blastocyst transfer. A subgroup analysis was performed to assess for potential heterogeneity of the hCG trigger effect on pregnancy outcomes. All data were analyzed using IBM SPSS 22.0 software (IBM Corp., Armonk, NY, USA). The level of significance was set at *P*<0.05.

## Results

### Baseline characteristics of the patients

**Figure 1:**
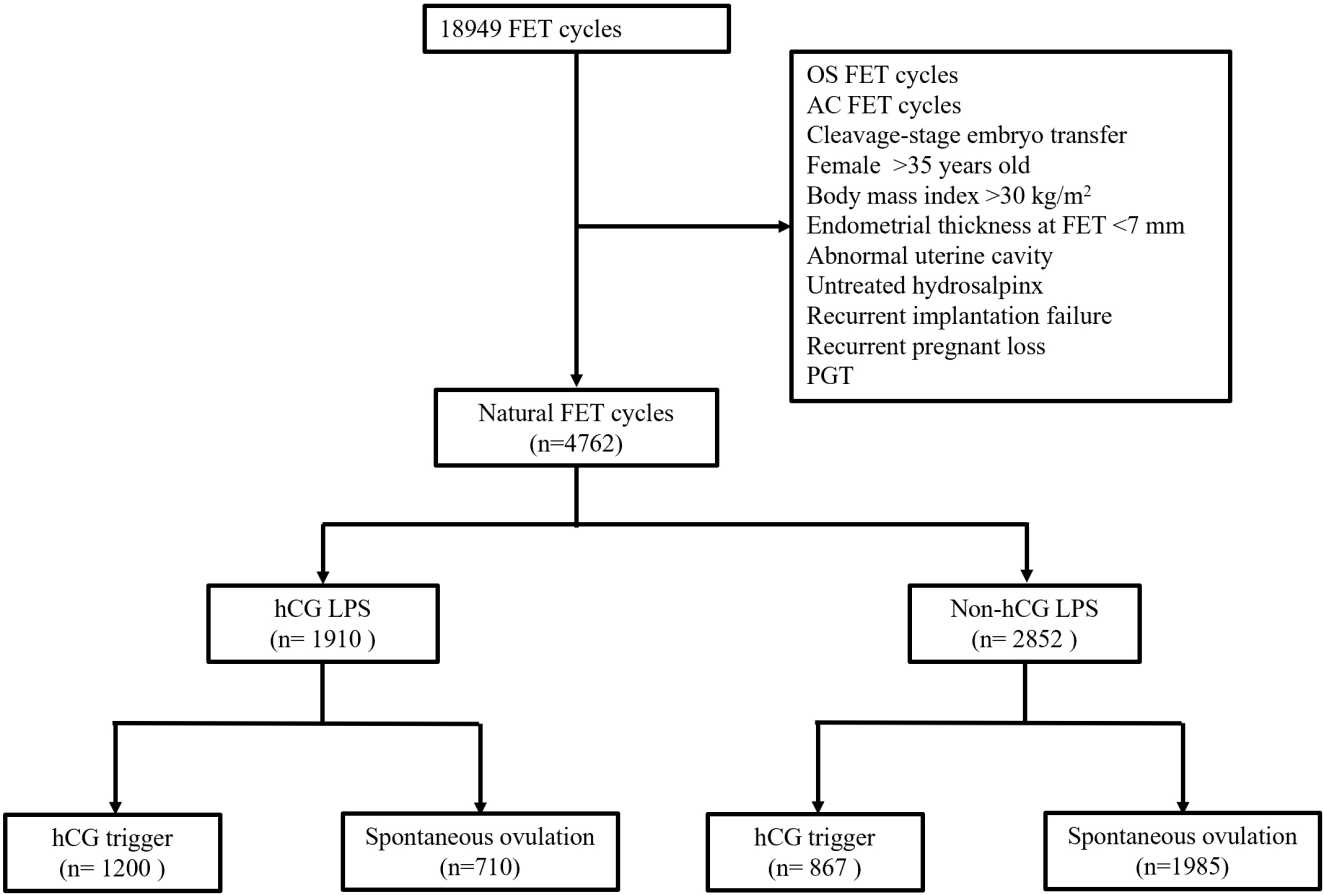
A flow chart of the studied population.

The flowchart of enrolled patients is shown in **Figure 1**. A total of 4762 women who fulfilled the inclusion and exclusion criteria were included in this study (**Table 1**). Of these, 1910 and 2852 patients were in the hCG LPS group and the non-hCG LPS group, respectively. Female age, male age, body mass index, infertility duration, parity, cause of infertility, basal FSH concentrations, and basal luteinizing hormone (LH) concentrations were similar in the two groups.

**Table 1:** Patient demographics of the study cohort.

	hCG LPS group (n=1910)	Non-hCG LPS group (n=2852)	*P* value
Female age at OPU (y)	30.75 ± 3.83	30.62 ± 3.71	0.241
Female age at FET (y)	31.73 ± 3.68	31.63 ± 3.59	0.340
Male age (y)	33.17 ± 4.28	33.13 ± 4.31	0.748
BMI, kg/m^2^	21.85 ± 2.73	21.76 ± 2.89	0.322
Years of infertility	3.39 ± 2.41	3.37 ± 2.44	0.731
Parity			0.776
Primipara, n (%)	82.04 (1567/1910)	82.36 (2349/2852)	
Multipara (%)	17.96 (343/1910)	17.64 (503 /2852)	
Type of infertility			0.251
Primary infertility, n (%)	52.51 (1003 /1910)	54.21 (1546 /2852)	
Secondary infertility, n (%)	47.49 (907 /1910)	45.79 (1306 /2852)	
Cause of infertility			0.960
Tubal factor, n (%)	39.01 (745/1910)	39.13 (1116 /2852)	
Ovulation factor, n (%)	4.66 (89/1910)	2.91 (83 /2852)	
Male factor, n (%)	15.55 (297 /1910)	18.34 (523 /2852)	
Combined factors, n (%)	36.39 (695/1910)	35.06 (1000 /2852)	
Unknown factor, n (%)	4.40 (84/1910)	4.56(130/2852)	
Basal FSH	7.22 ± 2.90	7.16 ± 2.72	0.509
Basal LH	5.44 ± 15.39	5.22 ± 6.12	0.498
No. of oocytes retrieved (n)	12.08 ± 5.68	12.76 ± 5.96	<0.001
Insemination type			0.151
IVF, n (%)	78.85(1506/1910)	77.14 (2200/2852)	
ICSI, n (%)	21.15(404/1910)	22.86 (652/2852)	
Freeze all, n (%)	45.50(869/1910)	45.65(1302/2852)	0.916
Endometrial thickness (mm)	11.30 ± 1.84	11.16 ± 2.61	0.038
hCG trigger, n (%)	62.83 (1200/1910)	30.40 (867/2852)	<0.001
Total No. of blastocysts transferred (n)	2344	3595	
Mean No. of blastocysts transferred (n)	1.23 ± 0.42	1.26 ± 0.44	0.009
Good quality blastocyst transfer			<0.001
None, n (%)	52.04 (994/1910)	44.60 (1272/2852)	
≥1, n (%)	47.96 (916/1910)	55.40 (1580/2852)	
Type of blastocyst transfer			0.432
Day 5 blastocyst, n (%)	88.44 (2073/2344)	87.76 (3155/3595)	
Day 6 blastocyst, n (%)	11.56 (271 /2344)	12.24 (440/3595)	

OPU, Oocyte pick up; FET, Frozen-thawed embryo transfer; FSH, Follicle stimulating hormone; LH, Luteinizing hormone. Data are presented as mean ± SD or n (%); Statistical significance is defined as P<0.05.

With regard to *in vitro* fertilization characteristics, the number of oocytes retrieved in the hCG LPS group was significantly greater than that in the non-hCG LPS group (12.08 ± 5.68 vs 12.76 ± 5.96, *P*<0.001). There was no significant difference in the insemination method or “freeze all” ratio between the groups.

With regard to FET characteristics, the rate of administration of a trigger in the hCG LPS group was 62.3%, which was twice that in the non-hCG LPS group (30.4%, *P*<0.001). In the hCG LPS group, the average number of blastocysts transferred was significantly less than that in the non-hCG LPS group (1.23 ± 0.42 vs 1.26 ± 0.44, *P*=0.009). Additionally, the rate of transferring good quality blastocyst transfer was significantly lower than that in the non-hCG LPS group (47.96% vs 55.4%, *P*<0.001). The type of blastocyst transfer was similar in the two groups.

### Pregnancy outcomes

The clinical pregnancy rate in the hCG LPS group was significantly lower than that in the non-hCG LPS group (63.82% vs 66.41%, aOR 0.872, 95% CI 0.765–0.996, *P*=0.046, **Table 2**). The live birth rate in the hCG LPS group was significantly lower than that in the non-hCG LPS group (53.98% vs 57.15%, aOR 0.873, 95% CI 0.766–0.991, *P*=0.035). The rates of biochemical pregnancy, implantation, twin pregnancy early pregnancy loss, and total pregnancy loss were not different between the groups (all *P*≥0.05).

**Table 2:** The comparison of IVF characteristics and outcomes.

	hCG LPS group (n=1910)	Non-hCG LPS group (n=2852)	Adjusted OR	95% CI	*P* value
Biochemical pregnancy, n (%)	69.37 (1325/1910)	70.41 (2008/2852)	0.948	0.821,1.080	0.382
Implantation rate, n (%)	59.64 (1398/2344)	60.78 (2185/3595)	1.100	0.979,1.235	0.095
Clinical pregnancy, n (%)	63.82 (1219/1910)	66.41 (1894/2852)	0.872	0.765,0.996	0.046
Twin pregnancy, n (%)	9.11(111/1219)	11.25(213/1894)			
Early pregnancy loss, n (%)	11.07 (135/1219)	10.24(194/1894)	1.081	0.840,1.388	0.560
Total pregnancy loss, n (%)	14.27 (174/1219)	12.88 (244/1894)	1.105	0.881,1.386	0.387
Live birth, n (%)	53.98 (1031/1910)	57.15 (1630/2852)	0.873	0.766,0.991	0.035

Adjusted: Female age, endometrial thickness, No. of blastocysts transferred, hCG trigger administration and good quality blastocyst transfer.

### Subgroup analysis

We performed a subgroup analysis by taking into consideration the potential effect of an hCG trigger on reproductive outcomes (**Table 3**). Among the 2067 women triggered with hCG, 1200 (58.06%) received hCG LPS and 867 (41.94%) did not receive hCG LPS. In women who were triggered with hCG, there was no significant differences in the rate of biochemical pregnancy, clinical pregnancy, early miscarriage, total miscarriage, or live birth between the two subgroups (all *P*≥0.05).

**Table 3:** Subgroup analysis of hCG LPS on FET outcomes.

	hCG trigger (n=2067)					Spontaneous ovulation (n=2695)				
	hCG LPS group (n=1200)	Non-hCG LPS group (n=867)	OR	95% CI	*P* value	hCG LPS group (n=710)	Non-hCG LPS group (n=1985)	OR	95% CI	*P* value
Biochemical pregnancy, n (%)	70.00(840/1200)	70.82(614/867)	0.980	0.801,1.180	0.753	68.31(485/710)	70.23(1394/1985)	0.912	0.765,1.095	0.311
Clinical pregnancy, n (%)	65.50 (786/1200)	66.67 (578/867)	0.967	0.811,1.169	0.776	60.99 (433/710)	67.21 (1316/1985)	0.784	0.652,0.946	0.011
Early miscarriage, n (%)	10.43 (82/786)	10.73 (63/587)	0.926	0.632,1.324	0.680	12.24 (53/433)	9.95 (131/1316)	1.240	0.874,1.758	0.227
Total miscarriage, n (%)	13.61 (107/786)	13.97 (82/587)	0.946	0.690,1.295	0.729	15.47 (67/433)	12.31 (162/1316)	1.289	0.939,1.769	0.116
Live birth, n (%)	56.00 (672/1200)	56.06 (486/867)	1.014	0.849,1.131	0.861	50.56 (359/710)	57.63 (1144/1985)	0.743	0.619,0.878	0.001

Adjusted: Female age, endometrial thickness, No. of blastocysts transferred and good quality blastocyst transfer.

However, among the 2695 women who ovulated spontaneously, 710 (26.35%) were treated with hCG LPS, and 1985 (73.65%) were not treated with hCG LPS. The rates of clinical pregnancy (60.99% vs 67.21%, aOR 0.786, 95% CI 0.652–0.946, *P*=0.011) and live birth (50.56% vs 57.63%, aOR 0.743, 95% CI 0.619–0.878, *P*=0.001) in the hCG LPS group were significantly lower than those in the non-hCG LPS group. The rate of biochemical pregnancy, early miscarriage, or total miscarriage was not different between the two groups (all *P*≥0.05).

## Discussion

The major finding of this study was that the clinical pregnancy and live birth rates in the hCG LPS group were significantly lower than those in the non-hCG LPS group (controls). Continuous hCG supplementation as LPS in natural FET cycles was associated with worse reproductive outcomes. In the subgroup analysis, in women who ovulated spontaneously, the clinical pregnancy and live birth rates in the hCG LPS group were significantly lower than those in the non-hCG LPS group. In women who received an hCG trigger, there was no significant difference in the clinical pregnancy rate or live birth rate between the hCG LPS group and the non-hCG LPS group.

There is some heterogeneity in published studies that examined the use of hCG for LPS. The dose and frequency differed greatly between the different studies, as well as the methods used for detecting ovulation and the use of hCG for triggering ovulation. In 2017, Lee et al. (12) reported that the use of hCG in natural FET cycles did not improve the ongoing pregnancy rate. In their RCT, women in the treatment group received 1500 IU hCG on the day of FET and 6 days after FET. The ongoing pregnancy rate, implantation rate, and miscarriage rate in the treatment group were similar to those in the control group. In 2019, another placebo-controlled RCT reported the same conclusion, although the method of hCG administration was different from previous published RCTs (15). Madani et al. treated women with 10,000 IU urinary hCG to trigger ovulation and 2500 IU hCG every 3 days after embryo transfer. They found that equally effective in terms of implantation, pregnancy, miscarriage, and live birth rates in women with and without hCG LPS. Cleavage-stage embryos were transferred in both studies.

Our study has some differences from previous published studies. First, we recruited women with only blastocyst transfer cycles. The number of blastocyst culture cycles is increasing worldwide owing to the development of embryo culture technology. The rate of implantation and the clinical pregnancy rate of blastocyst-stage embryos are much higher than those of cleavage-stage embryos (16). Therefore, we hypothesize that the effect of hCG LPS on outcomes of blastocyst transfer may be different from those of cleavage-stage embryo transfer. However, our results are in accordance with previous reports, which was unexpected. Second, we treated women in the hCG LPS group with continuous hCG injection after blastocyst-stage embryo transfer (2000 IU daily) for 5 days. Additionally, an hCG trigger was provided for ovulation when transvaginal ultrasound showed anovulation. There is a controversy regarding whether exogenous hCG will result in luteal phase deficiency through a short-loop feedback mechanism (17, 18). Continuous injection of hCG for LPS has been reported to be beneficial (19, 20). However, it is important to note that the hCG used in these studies was administered at a micro dose (100 IU) and the cycles involved fresh embryo transfer. Therefore, it is not certain that the same benefits would be observed in frozen embryo transfer (FET) cycles. In China, 2000 IU and 5000 IU hCG injections are the most common. Taking into consideration that T_max_ of an hCG injection is 12 h and T_1/2_ is 23 h, we continuously treated our patients with 2000 IU hCG injections qd. Intramuscular hCG has a long half-life. Intramuscular hCG achieves circulating serum concentrations for approximately 7 days after administration. Therefore, we added hCG injection for 5 days after blastocyst FET. Third, we monitored ovulation by frequent checking of transvaginal ultrasound and urine LH. Other studies timed ovulation by the serum LH surge. Fourth, we combined hCG with oral and vaginal progesterone for luteal support, while in previous reports (12), only hCG was used as luteal support. The effect of hCG LPS on reproductive outcomes may have been attenuated by other progesterone that we used simultaneously.

Luteal support was beneficial following natural FET cycles, especially progesterone administration. Although hCG could enhance endogenous production of progesterone by the corpus luteum, supraphysiological levels of hCG supplementation did not improve reproductive outcomes.

The effects of an hCG trigger on reproductive outcomes are conflicting (21–23). An hCG trigger can cause an early rise of progesterone and finally lead to advancement of the endometrium and reduced implantation (24). We performed a subgroup analysis to assess the potential heterogeneity of an hCG trigger effect on pregnancy outcomes. In women who received hCG triggering, hCG LPS was not associated with any adverse effect on reproductive outcomes. However, in women who ovulated spontaneously, there was a significant decrease in the clinical pregnancy and live birth rates in women who received hCG LPS. This finding indicates that, in women who can ovulate on their own, extra hCG LPS is detrimental. A possible explanation for the adverse effect of continuous hCG LPS is as follows, in women who can ovulate spontaneously, their pituitary can secrete sufficient LH, and their LH surge can be detected in their serum and urine (17). The administration of continuous external hCG may directly inhibit LH release via negative feedback actions at the hypothalamic–pituitary level. The potential for increasing serum hCG concentrations by augmenting their stimulation with hCG is limited by its capacity. In women in whom an LH surge could not be detected, exogenously added hCG did not cause any harm to their defective pituitary secretion of LH. However, the mechanism of this finding requires further research.

The major strength of our study is that we focused on natural blastocyst FET cycles, eliminating the effects of the developmental stage on reproductive outcomes. There are several limitations to our study. First, this was an observational study, and there was a lack of information regarding the reasons for supplementation of hCG as LPS. Although we used a multivariable logistic regression to control for confounders between the two groups, the findings of our study might have been confounded by unmeasured or unidentified covariates. Second, all data of *in vitro* fertilization treatment were from a single center. Third, we did not measure serum hormones after blastocyst FET, which meant that we were unable to determine the reason for the decreased clinical pregnancy and live birth rates in the hCG LPS group.

## Conclusion

Our study suggests that hCG LPS is non-beneficial in women who receive natural FET cycles with blastocyst transfer. Additionally. the adverse effect of hCG LPS is more pronounced in women who receive no hCG trigger. Further research is required to better understand the mechanisms behind the association.

## Data Availability

The original contributions presented in the study are included in the article/Supplementary Material. Further inquiries can be directed to the corresponding author.
